# Motor cortex excitability and inhibitory imbalance in autism spectrum disorder assessed with transcranial magnetic stimulation: a systematic review

**DOI:** 10.1038/s41398-019-0444-3

**Published:** 2019-03-07

**Authors:** Fumi Masuda, Shinichiro Nakajima, Takahiro Miyazaki, Kazunari Yoshida, Sakiko Tsugawa, Masataka Wada, Kamiyu Ogyu, Paul E. Croarkin, Daniel M. Blumberger, Zafiris J. Daskalakis, Masaru Mimura, Yoshihiro Noda

**Affiliations:** 1 Department of Neuropsychiatry, Keio University School of Medicine, Tokyo, Japan; 2 Department of Psychiatry, Shiga University of Medical Science, Shiga, Japan; 3 Multimodal Imaging Group, Centre for Addiction and Mental Health and Department of Psychiatry, University of Toronto, Toronto, ON Canada; 4 Department of Psychiatry and Psychology, Mayo Clinic, Rochester, MN USA; 5 Pharmacogenetics Research Clinic, Centre for Addiction and Mental Health, Toronto, ON Canada; 6 Temerty Centre for Therapeutic Brain Intervention, Centre for Addiction and Mental Health and Department of Psychiatry, University of Toronto, Toronto, ON Canada

## Abstract

Cortical excitation/inhibition (E/I) imbalances contribute to various clinical symptoms observed in autism spectrum disorder (ASD). However, the detailed pathophysiologic underpinning of E/I imbalance remains uncertain. Transcranial magnetic stimulation (TMS) motor-evoked potentials (MEP) are a non-invasive tool for examining cortical inhibition in ASD. Here, we conducted a systematic review on TMS neurophysiology in motor cortex (M1) such as MEPs and short-interval intracortical inhibition (SICI) between individuals with ASD and controls. Out of 538 initial records, we identified six articles. Five studies measured MEP, where four studies measured SICI. There were no differences in MEP amplitudes between the two groups, whereas SICI was likely to be reduced in individuals with ASD compared with controls. Notably, SICI largely reflects GABA(A) receptor-mediated function. Conversely, other magnetic resonance spectroscopy and postmortem methodologies assess GABA levels. The present review demonstrated that there may be neurophysiological deficits in GABA receptor-mediated function in ASD. In conclusion, reduced GABAergic function in the neural circuits could underlie the E/I imbalance in ASD, which may be related to the pathophysiology of clinical symptoms of ASD. Therefore, a novel treatment that targets the neural circuits related to GABA(A) receptor-mediated function in regions involved in the pathophysiology of ASD may be promising.

## Introduction

Autism spectrum disorder (ASD) is a neurodevelopmental disorder with impairments in social interaction and communication, accompanied with restricted and repetitive behaviors^1^. Although little is known about the neurobiological mechanisms underlying the symptoms of ASD, impaired cortical inhibitory control might explain some of the symptoms. Previous studies have shown that the E/I imbalance could impair sensory, mnemonic, social, emotional, and other forms of neurocognitive function, depending on the affected neural network^2,3^. As these symptoms are commonly found in ASD, impaired cortical inhibition could explain the pathophysiology of this neurodevelopmental disorder^4–6^. For example, recent studies reported reduced inhibitory control in a Go-No-Go task in adults with ASD compared with controls^7–9^.

Specifically, over 90% of individuals with ASD display more than one type of sensory dysfunction with extreme levels such as hypersensitivity^10^, which may stem from the inhibitory dysfunction on sensory inputs. Furthermore, impaired inhibition might also account for some of the difficulties in social communication in ASD such as “aloof”, “passive”, and “active-but-odd” attitudes^11,12^. For example, children with “active-but-odd” characteristics tend to seek interactions with others actively, albeit in an unusual way^13^, possibly owing to an neurophysiological excitation/inhibition (E/I) imbalance and manifestation of clinical symptoms such as sensory deficits and/or social communication deficits in individuals with ASD^14–16^.

The E/I balance is assumed to be continuously controlled by the interaction of gamma-aminobutyric acid (GABA)ergic and glutamatergic functions. Especially, GABA receptor-mediated dysfunction has been proposed as a pathological hypothesis in ASD, which posits that the clinical symptoms including a deterioration in the quality of sensory information may be caused by the failure to suppress competing “noise”^17^. Indeed, prepulse inhibition, involving the sensorimotor gating through GABAergic neurotransmission^18,19^, is impaired in adults with ASD^20,21^. Furthermore, previous studies showed decreased gamma-band oscillation activity in the temporal area evoked by a pure tone in individuals with ASD compared with healthy controls^22,23^, which may represent reduced activity of inhibitory GABAergic interneurons in ASD^24,25^.

From a neurochemical point of view, magnetic resonance spectroscopy (MRS) studies showed reduced GABA levels in the anterior cingulate cortex, left auditory cortex, and left motor cortex (M1) of individuals with ASD compared with controls^26–30^. In addition, reduced GABA levels in the anterior cingulate cortex in individuals with ASD were correlated with greater severity of clinical symptoms, such as social cognition^26^, inference emotional state^26^, motor stereotypies^31^, or self-assessment^32^. In postmortem studies, GABA(A) receptors were reduced in the parietal, cerebellar, and frontal regions in individuals with ASD in comparison with healthy controls^33,34^. Collectively, these findings suggest that dysfunction of GABA receptor in the neural circuits may be associated with the pathophysiology of ASD^2,35,36^.

However, although these results show static findings of substance level of GABA, there is a limit that it does not clarify the physiological dynamics of GABA. Therefore, transcranial magnetic stimulation (TMS) is a useful technique for overcoming the limit, and it enables the exploration of the dynamic functional properties of neural circuits non-invasively. With this technique, we can differentially examine the function of the neural circuits that involves various neurotransmitter associated with cortical functions at the targeted area in human subjects in health or neurological and psychiatric conditions^37^. A recent systematic review and meta-analysis focused on the excitability of M1 suggested a lower cortical inhibition in individuals with attention deficit/hyperactivity disorders (ADHD), another major class of neurodevelopmental disorder, compared with controls^38^. Thus, TMS may be a promising tool to assess the neurophysiological basis represented by the E/I balance of ASD. Indeed, several established paradigms are available in TMS research to assess different properties of the neural circuits, for example, motor-evoked potential (MEP), short-interval intracortical inhibition (SICI), intracortical facilitation (ICF), long-interval intracortical inhibition (LICI), and paired associative stimulation (PAS). MEP reflects the excitability of the membrane potential of pyramidal neurons in M1^39^. SICI largely reflects GABA(A) receptor-mediated inhibitory function^40–42^. SICI is assumed to be induced by through the conditioning stimulus that activates a low threshold inhibitory system, which in turn suppresses the inhibitory interneurons by hyperpolarizing inhibitory postsynaptic potentials, resulting in an inhibition of the cortical output evoked by a subsequent test stimulus^40,42,43^. On the other hand, ICF is assumed to represent excitatory transmission largely through the glutamatergic *N*-methyl-d-aspartate receptor^44–46^, which is thought to be evoked by the summation of excitatory postsynaptic potentials^47,48^.

To date, neurochemical studies by MRS and postmortem brain research suggest the abnormal E/I balance involvement in neural circuits at various brain areas in individuals with ASD^3,33,49^; however, in terms of the TMS neurophysiology, there is still no clear consensus on whether the E/I balance is biased toward excitation or inhibition in the involved neural circuits in ASD. Therefore, we conducted a systematic review on TMS neurophysiology including MEP, SICI, and ICF to identify whether the E/I balance is altered in the involved areas of brain in individuals with ASD.

## Methods

### Search strategy

This systematic review was performed according to the Preferred Reporting Items for Systematic Reviews and Meta-Analyses (PRISMA) guidelines^50^. Electronic databases (PubMed, Medline, Embase, and PsycINFO) were searched up to 27th April 2018 using the terms related to stimulation to the cortex (“non-invasive brain stimulation”, “TMS”, and “transcranial magnetic stimulation”), those related to assessment of neurophysiology (“brain activity”, “brain waves”, “EEG”, “electrocorticography”, “electroencephalogram(s)”, “electroencephalography”, “EMG”, “magnetoencephalography”, “MEG”, “MEP”, “motor-evoked potential”, “neurophysiolo”, “neuroplasticity”, “plasticity”, and “plastic”), and those related to neurodevelopmental disorders especially ASD (“ADHD”, “ASD”, “asperger”, “attention deficit hyperactivity disorder”, “autism”, “autistic”, “childhood schizophrenia”, “developmental disorder”, “neurodevelopmental disability”, “neurodevelopmental disorder”, and “PDD”). We included “ADHD” as one of search terms, as individuals with ASD often have comorbid diagnosis of ADHD. Two authors (F.M. and Y.N.) independently searched and assessed eligibility, and further extracted data.

#### Study selection

Studies were included if articles met the following inclusion criteria: (i) written in English; (ii) involved participants diagnosed with ASD by DSM-IV, DSM-IV-TR, or ADOS-2 and controls; and (iii) measured with TMS neurophysiology such as MEP and SICI. In contrast, articles were excluded according to the following criteria: (iv) animal model studies; (v) review articles; or (vi) case reports.

#### Outcome measures

The outcome measures were TMS neurophysiology regarding MEP, SICI, ICF, LICI, and PAS.

#### Quality assessment

The Risk of Bias Assessment tool for Nonrandomized Studies was employed^51^ for the following factors: participant selection, confounding variables, measurement of exposure, blinding of outcome assessment, incomplete outcome data, and selective outcome reporting.

#### Data extraction

Data relevant to the research purpose were extracted from each article as follows: (1) characteristics of participants; (2) outcome measures; (3) main results; and (4) mean values and standard deviations for the different excitability parameters. If there was a possibility that the subjects were duplicated among the extracted studies, we asked the author and confirmed the presence or absence of duplication of the subjects. In the present study, we performed preliminary meta-analyses on the above TMS neurophysiological indices and summarized them as supplementary materials.

## Results

**Fig. 1:**
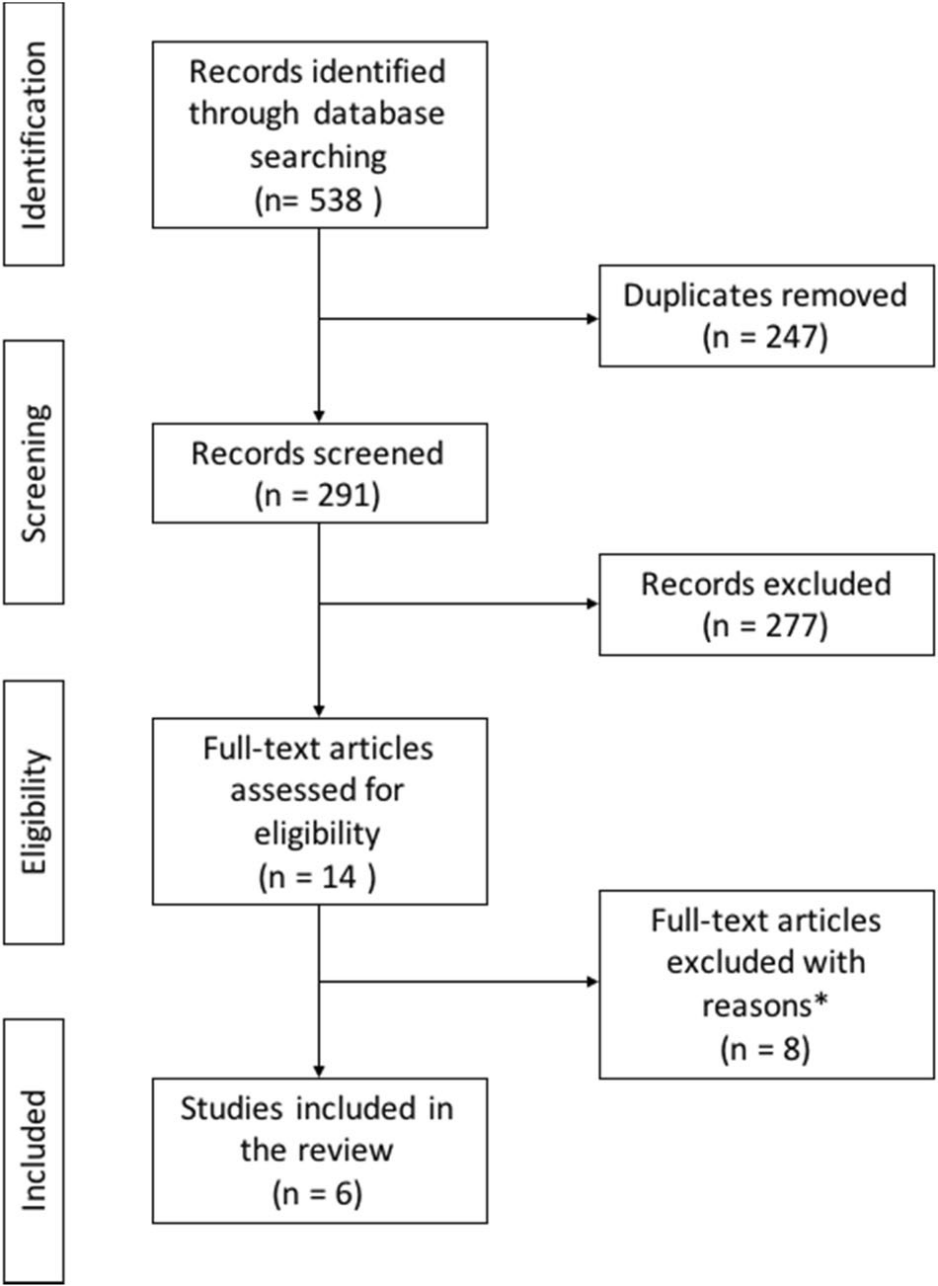
Preferred reporting items for systematic reviews and meta-analyses (PRISMA) flow diagram

The selection process in this systematic review is shown in Fig. 1. The search identified 538 articles, six of which met eligibility criteria. All of the included studies are summarized in Table 1 with extracted data such as study population, investigated measures, and each value of parameters. Among them, five studies measured MEP^4,52–55^, four studies measured SICI^4,53,54,56^, two studies measured LICI^54,56^, one study measured ICF^4^, and one study measured PAS^53^. There were no studies in which subjects clearly overlapped among the extracted studies. Results of quality assessment such as risk of bias are summarized in Supplementary Figure 1. Further, the funnel plots are displayed in Supplementary Figures 4 and 5.

**Table 1:** Characteristics of the included studies between individuals with ASD and controls

Authors, Year	Study population (male/female)	Age (mean ± SD) (years)	Age range (years)	Medication	Investigated measures	Local of investigation	MEP (mV mean (SD))	SICI (mean (SD))	Other parameters	Main findings and interpretation
I. Minio-Paluello, et al. 2009	Patients (16M)	28.0 ± 7.2	Unknown	Not listed	MEP	Left M1/right FDI	1.84 (0.68)	–	–	No difference in MEP.
	Controls (20M)	25.3 ± 6.7					1.54 (1.24)			
P.G. Enticott, et al. 2010	Patients (20M/5F)	18.1 ± 4.4	Unknown	+	MEP, SICI, ICF	Left and right motor cortex/left and right APB	0.67 (0.58)	0.66 (0.49)	ICF: 1.94 (0.40)	SICI was higher in individuals with ASD: cortical inhibition was reduced in individuals with ASD. No difference in MEP and ICF.
	controls (8M/3F)	19.0 ± 3.1					0.60 (0.40)	0.46 (0.28)	1.59 (0.91)	
L. Oberman, et al. 2010	Patients^5^	40.8 ± 10.7	26–54	Not listed	SICI, LICI	Left motor cortex/right FDI	–	1.11 (1.43)	LICI: 0.54 (0.82)	No difference in SICI and LICI.
	controls^5^	38.6 ± 13.8	22–54					0.28 (0.06)	0.15 (0.12)	
P.G. Enticott, et al. 2013	Patients (28M/8F)	26.0 ± 10.5	Unknown	+	MEP, SICI, ICF	Left and right M1/left and right FDI	1.80 (1.74)	0.62 (0.39)	LICI: 0.22 (0.28)	No difference in MEP, SICI, and LICI.
	controls (23M/11F)	26.2 ± 6.6					1.86 (2.03)	0.49 (0.20)	0.27 (0.40)	
N.H. Jung, et al. 2013	Patients (14M/1F)	17.1 ± 4.5	15–29	–	MEP, SICI, PAS	Right M1/non-dominant APB	0.95 (0.11)	0.42 (0.24)	MEP (0 min after post PAS):	PAS effect on MEP was significant in controls at time points 60 min compared with individuals with ASD (controls > individuals with ASD): neural plasticity induced by PAS was reduced in individuals with ASD. No difference in MEP and SICI.
	controls (5 M/4 F)	22.4 ± 5.2	Unknown				1.00 (0.14)	0.40 (0.23)	1.07 (0.28)	
									1.26 (0.47)	
									MEP (30 min after post PAS):	
									0.99 (0.47)	
									1.64 (0.92)	
									MEP (60 min after post PAS):	
									0.97 (0.37)	
									1.71 (0.61)	
E.V. Pedapati, et al. 2016	Patients (7M/2F)	15.6 ± 1.8	13–18	+	MEP	Dominant M1/dominant FDI	1.9 (1.12)	–	–	No difference in MEP.
	controls (5M/4F)	14.5 ± 2.2	11–18				2.8 (1.7)			

*In seven articles, no results measured with TMS neurophysiology such as MEP or SICI was available. One article had a sample that overlapped with another research study

### MEP

Five out of the six studies measured MEP^4,52–55^ (Table 1). All of the five studies identified no differences between individuals with ASD (a total of 101 individuals, age: 22.1 ± 8.8 yrs.) and controls (a total of 83 individuals, age: 23.3 ± 6.9 yrs.). Two studies investigated MEP on FDI muscle^54,56^, other two studies did on APB muscle^4,53^, and the other one study did both on FDI and APB muscles^52^. The MEP values measured on FDI muscle is likely to be higher compared with measure on APB.

### SICI

Four out of the six studies measured SICI in individuals with ASD (a total of 81 individuals, age: 22.8 ± 10.1 yrs.) and controls (a total of 59 individuals, age: 25.3 ± 8.2 yrs.)^4,53,54,56^ from the M1 (Table 1). One study showed that individuals with ASD had significantly reduced SICI compared with controls^56^, whereas other three studies showed no differences in SICI between the two groups^4,53,54^. Enticott et al. distinguished autism group to the two group: “high-functioning autism” and “Asperger group”^4^. This study showed that SICI was significantly reduced in the high-functioning autism group compared with both the Asperger disorder group and neurotypical group. There was no significant difference between the Asperger disorder group and neurotypical group. In addition, Enticott et al.^54^ showed ASD with early language delay group had significantly reduced SICI compared with both ASD without language delay group and neurotypical group.

### Other TMS metrics of LICI, ICF, and PAS

Both the two studies on LICI^54,56^ showed no differences in LICI between the two groups. The one study on ICF^4^ noted no difference in ICF between the two. Lastly, the one PAS study demonstrated that MEP amplitude in controls was significantly increased at time points of 30 min and 60 min after PAS compared with baseline^53^, while there was no significant increase in MEP amplitude in individuals with ASD. Although there was no difference in PAS effect on MEP between the two groups at the time point of 30 min, PAS effect on MEP was significant in controls at the time point of 60 min compared with individuals with ASD (i.e., controls > individuals with ASD).

## Discussion

To our knowledge, this is the first systematic review to compare TMS neurophysiological metrics such as MEP and SICI between individuals with ASD and controls. This systematic review showed that SICI was likely to be reduced in individuals with ASD compared with controls with mild to moderate effect size, whereas there was no difference in MEP amplitude between the two groups.

First, there was no difference in MEP amplitudes between individuals with ASD and controls in each of studies. MEP amplitudes by TMS varied among studies, depending on the muscles of interest. Consistent with previous studies, first dorsal interosseous (FDI) muscle showed relatively higher MEP amplitude, whereas APB muscle showed relatively lower MEP amplitude^57,58^. In ASD, ion channels related to membrane potential such as calcium, sodium, and potassium deficits are to be associated with the underlying pathogenesis contributing to cortical inhibitory dysfunction^59^. Weiss et al.^60^ reported that only 5% of families carried the variants with potential effects on sodium channel function, which mainly relates to membrane potential. In general, it is known that MEP amplitude could be influenced by age, lesion location, motor deficit, and exercises^61–63^. However, the influence of autistic trait on MEP has not been elucidated yet. At least, based on the result of this review, the difference of the membrane potential excitability as indexed by MEP was not observed between the two groups might be masked in individuals with ASD.

Second, as SICI predominantly reflects GABA(A) receptor-mediated function^40–42^, the results of this review suggest that individuals with ASD may have GABA(A)ergic dysfunction, which is consistent with previous studies^17,26–28,33,34^. Furthermore, the degree of cortical inhibition impairment has been shown to be different depending on the subtype of ASD, such as autism with language delay and without delay^4,54^.

In animal studies, mice compromising the phospho-dependent regulation of the GABA(A) receptor β3 subunit manifests the core phenotypes of ASD such as increased repetitive behavior and decreased social interaction^64^, whereas mice lacking Mecp2 gene in the GABA-releasing neurons showed increased autistic features such as long interaction with an unfamiliar mouse compared with controls^65^. Indeed, a small percentage of individuals with ASD carry mutations in genes encoding neuroligins, which result in abnormality in postsynaptic cell-adhesion molecules^66^. Also, mice with autism-linked neuroligin-3 R451C mutation show impaired social interactions accompanied by an altered inhibitory synaptic transmission^66^. Further, the mutations in GABA(A) receptor subunit genes (i.e., GABRB3, GABRA5, and GABRQ) were identified in patients with ASD^36,67–69^.

As mentioned above, previous MRS studies showed decreased GABA levels in the anterior cingulate cortex, left auditory cortex, and left M1^26–28,33^, and postmortem studies showed reduced GABA(A) receptors in individuals with ASD compared with controls^33,34^. In addition, there are several MRS studies that demonstrated the positive relationship between GABA levels and severity of symptoms^26,31,32^. Cochran et al. showed that GABA/Cre levels in the ACC were positively correlated with the score of inference emotional state, intelligent quotient, and severity of social cognitive impairment in the individuals with ASD^26^. Harris et al.^31^ demonstrated that GABA/Cre levels in the ACC were significantly associated with greater severity of motor stereotypies in the individuals with ASD. Brix et al.^32^ found a significant negative correlation in the ASD group between the scores on the Autism Spectrum Screening Questionnaire (ASSQ) and GABA/Cre levels in the left ACC. Although there is no TMS–EEG study that proved the relationship between dynamic GABAergic function and clinical symptoms in ASD, the relationship would be highly expected in this disorder. Taken together, these findings suggest that GABA(A) receptor function as indexed by TMS–EEG can be a promising indicator of intervention and its modulation may be a novel potential treatment for ASD to improve their clinical symptoms. For example, there are some compounds currently under research that target the specific membrane transport protein, related to GABA(A) receptor-mediated function.

However, it is known that benzodiazepines, which are positive allosteric GABA(A) receptor modulators, can cause atypical responses such as anxiety, aggression, or hyperarousal in individuals with autism^70–72^. This phenomenon is explained by the chloride homeodynamics theory that high levels of intracellular chloride owing to an immature development of the membrane transport proteins, such as the Na-K-Cl cotransporter (NKCC1) and K-Cl cotransporter (KCC2), cause an outward chloride current from the GABA(A) receptor, which results in the alteration of nature of GABA(A) receptor to excitatory function^73,74^. Specifically, in immature neuron, abnormal down-regulation of the KCC2 as well as an upregulation of selective chloride importer, NKCC1, are observed^73,74^. Therefore, the NKCC1 antagonist, bumetanide, is suggested to have beneficial impact by inducing indirect on the symptoms of ASD^75–77^. Furthermore, in ASD model mouse, α2,3 subunit-selective positive allosteric modulator L-838417 was effective, whereas α1-subunit-selective drug zolpidem exacerbated social deficits^78^. Thus, pharmacological treatment modifying the function of GABA(A) receptor subunits function may contribute to the improvement of the symptoms of ASD.

On the other hand, glutamate, the neurotransmitter involved in the excitatory component of the E/I balance, is suggested to be increased in individuals with ASD^26,33,79–81^. Indeed, postmortem studies have discovered the structural and functional changes in both glutamatergic excitatory and GABAergic inhibitory circuits in individuals with ASD^80,81^. Further, MRS studies have indicated that glutamate levels in striatum, anterior cingulate cortex, left cerebellar, and left frontal cortex were increased in individuals with ASD compared with controls^26,33,79^. Collectively, these results suggested that individuals with ASD may have increased cortical excitation in the involved area of brain. Together with the result of SICI, individuals with ASD may have the E/I balance biased toward excitation, as a result of reduced inhibition and/or increased excitation.

In addition, several genetic mutations have been reported in relation to synaptic formation in individuals with ASD such as NLGNs, SHANKs, and PTEN for dendritic spines of pyramidal cells, and NRXNs and FMRP for parvalbumin interneurons like basket cells and chandelier cells^80,82^. The mutations of these genes could cause impaired synapse formation and synaptic pruning, which may lead to the E/I imbalance in ASD^83,84^. These abnormalities in synaptic proteins may be linked to the E/I imbalance in individuals with ASD via altered neural system that involved GABA receptor-mediated function as suggested by the SICI studies on ASD.

However, there are several studies that are inconsistent with previously determined direction of possible E/I balance bias in ASD^85–88^. Contrary to our finding, several MRS studies reported that decreased excitation in the frontal lobe, occipital lobe, and anterior cingulate cortex in individuals with ASD compared with controls^33,85,86^. Further, perceptual measures as assessed by the color discrimination or pure pitch discrimination showed increased inhibition in individuals with ASD^87,88^. In clinical practice, altered responsiveness to sensory input toward both the opposite directions are assessed as either hyposensitivity as known as “low registration” or hypersensitivity as “sensory sensitivity”^89^. Although these symptoms look inconsistent, they often co-exist. Therefore, the assessment of E/I balance from different brain regions may be needed to explain the inconsistent symptoms of ASD. Therefore, the present findings warrant further TMS neurophysiological studies with larger sample sizes that include other paradigms such as ICF, LICI, short-latency afferent inhibition, and PAS to elucidate comprehensive understanding of the neural basis of ASD.

### Limitation

The limitation of this systematic review is the small sample size for each parameter. Also, each study included a limited number of individuals, which did not allow us to control the confounding effects of the age, gender, accompanied medication, or comorbidity such as other neurodevelopmental disorders. Further, in this study, because of the limited number of articles investigating the non-motor areas in ASD, only M1 could be systematically reviewed.

### Future direction

Further well-designed studies addressing current limitations and the knowledge gap are warranted. At present, our knowledge is quite limited about the neurobiological mechanism of ASD, although it is the key for establishing methods for precise diagnosis and efficient treatment^90^. Among various paradigms in TMS study, PAS paradigm might be the promising paradigm to seek for. Although we found only one study that measured PAS in this review, PAS paradigm could index the degree of neuroplasticity in motor as well as non-motor areas in individuals with ASD, and thus be useful in assessing the E/I balance in the neural circuits at the targeted brain area. Therapeutic intervention with brain stimulation might also prove beneficial as a novel treatment tool for ASD, for example, by applying repetitive TMS or theta-burst stimulation to readjust E/I balance in specific brain areas^91,92^. By combining the two aspects of TMS as a tool to probe and modulate the neural function, it may even become possible to implement a tailor-made treatment that optimizes therapeutic outcomes in the future, applying appropriate stimulation to the brain regions involved in this disorder.

## Conclusions

This systematic review demonstrated reduced cortical inhibition of M1 in individuals with ASD with no difference of MEP compared with controls. This finding is in line with the converging evidence that suggests altered E/I balance in ASD. Given that impaired GABAergic and/or glutamatergic systems could cause the E/I imbalance in the neural circuits, the present systematic review warrant further TMS studies in ASD with larger sample sizes and more-comprehensive neurophysiological and neuropsychological measures. Targeting a wider range of brain areas outside of M1—dorsolateral prefrontal cortex—which would be more closely related to cognitive and/or sensory functions of the disorder is the next logical area of investigation.

## Supplementary information

Supplemental legends

Supplementary Material.

Supplementary Figure 1.

Supplementary Figure 2.

Supplementary Figure 3.

Supplementary Figure 4.

Supplementary Figure 5.

Supplementary Figure 6.

Supplementary Figure 7.
