# Supplementary material for: Motor cortex excitability and inhibitory imbalance in autism spectrum disorder assessed with transcranial magnetic stimulation: a systematic review
Source: Transl Psychiatry. 2019 Mar 7;9:110. doi: 10.1038/s41398-019-0444-3 (PMC6405856; doi:10.1038/s41398-019-0444-3)
Supplement: Supplementary file 1 — Supplemental legends [file 41398_2019_444_MOESM1_ESM.docx]

Supplementary Material.

Methods and results for preliminary meta-analyses and meta-regression analyses

Supplementary Figure 1. Summary of the Risk of Bias for the included Studies.

Supplementary Figure 2. Group differences in MEP between individuals with ASD and controls.

Supplementary Figure 3. Group differences in SICI between individuals with ASD and controls.

Supplementary Figure 4. Meta-regression analyses examining the relationship among individual’s age and MEP amplitudes between individuals with ASD and controls.

Supplementary Figure 5. Meta-regression analysis examining the relationship among male ratio and MEP amplitudes between individuals with ASD and controls. Supplementary Figure 6. A funnel plot for MEP data in the included studies

Supplementary Figure 7. A funnel plot for SICI data in the included studies
