# Supplementary material for: Motor cortex excitability and inhibitory imbalance in autism spectrum disorder assessed with transcranial magnetic stimulation: a systematic review
Source: Transl Psychiatry. 2019 Mar 7;9:110. doi: 10.1038/s41398-019-0444-3 (PMC6405856; doi:10.1038/s41398-019-0444-3)
Supplement: Supplementary file 2 — Supplementary Material. [file 41398_2019_444_MOESM2_ESM.docx]

**Supplementary Material**

**Methods for preliminary meta-analyses and meta-regression analyses**

The main outcomes for individuals with ASD and controls from the included studies were used in the meta-analyses. The primary meta-analyses and meta-regression analyses were conducted using the Comprehensive Meta-Analysis (www.meta-analysis.com) and Review Manager 5.3 (http://tech.cochrane.org/revman) software. Each study parameter, MEP, and SICI were analyzed separately. Differences in MEP or SICI between individuals with ASD and controls were calculated using standardized mean difference (SMDs). SMD and two-sided 95% conﬁdence intervals (CI) were chosen as the summary statistic for the meta-analysis. Effect size of MEP and SICI between the two groups were calculated by dividing mean differences by weighted and pooled standard deviations (SDs). In cases where mean or SD values were not reported, we manually measured depicted mean and SD values from chart figures. Effect size was interpreted as follows: small, SMD = 0.2; medium, SMD = 0.5; and large, SMD = 0.8. Meta-regression was performed if parameter and participant demographics were available in ≥5 datasets. For the meta-regression, we used “age” or “male ratio” of individuals with ASD as independent variables.  Moreover, to adjust for study heterogeneity, the inverse variance statistical method and random effects model were employed. Significance level was set at two-sided 95% confidence intervals (CIs). The I2 statistic was applied to assess study heterogeneity. Specifically, I2 ≥ 50% represented significant heterogeneity.

**Result**

**1. MEP**

In a primary meta-analysis, MEP did not distinguish ASD (mean = 1.40, SD = 1.26) from controls (mean = 1.62, SD = 1.64) (**Supplementary Figure 2**). Further, in meta-regression analyses, subjects’ age or male ratio were not associated with SMDs between individuals with ASD and controls (**Supplementary Figure 4 and** **Supplementary Figure 5**). Medication data were insufficient in the included studies to perform a meta-regression analysis.

**2. SICI**

In the primary meta-analysis, ratio of SICI was significantly higher in individuals with ASD (mean = 0.63, SD = 0.52) compared to controls (mean = 0.45, SD = 0.22) (**Supplementary** **Figure 3**). However, four studies examining SICI were insufficient to perform meta-regression analyses.
