# Supplementary material for: Motor cortex excitability and inhibitory imbalance in autism spectrum disorder assessed with transcranial magnetic stimulation: a systematic review
Source: Transl Psychiatry. 2019 Mar 7;9:110. doi: 10.1038/s41398-019-0444-3 (PMC6405856; doi:10.1038/s41398-019-0444-3)

**Supplementary Figure 1.** Summary of the Risk of Bias for the Included Studies.


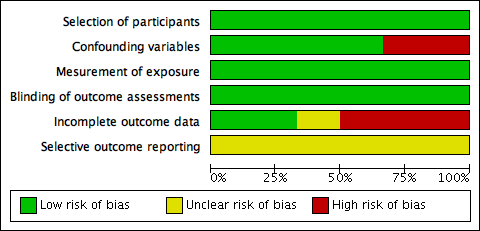


Risk of bias related to “selection of participants”, “measurement of exposure”, and “blinding of outcome assessment” were considered “low”. Individuals with ASD were diagnosed based on DSM-IV (four studies), DSM-IV-TR (one study), or ADOS-2 (one study). Risk of bias related to “confounding variables” was “high” for two studies, since there was no description about medication. Risk of bias related to “incomplete outcome data” was “high” for three studies, since there was no information about drop-out reasons. Risk of bias related to "selective outcome reporting" was “unclear” for all studies, since we could not obtain all of experimental protocols.


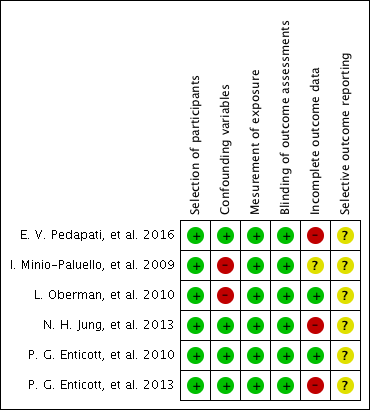

Supplement: Supplementary file 3 — Supplementary Figure 1. [file 41398_2019_444_MOESM3_ESM.docx]
