# Supplementary figures and images for: Motor cortex excitability and inhibitory imbalance in autism spectrum disorder assessed with transcranial magnetic stimulation: a systematic review
Source: Transl Psychiatry. 2019 Mar 7;9:110. doi: 10.1038/s41398-019-0444-3 (PMC6405856; doi:10.1038/s41398-019-0444-3)

**Supplementary Figure 2.** Group differences in MEP between individuals with ASD and controls.


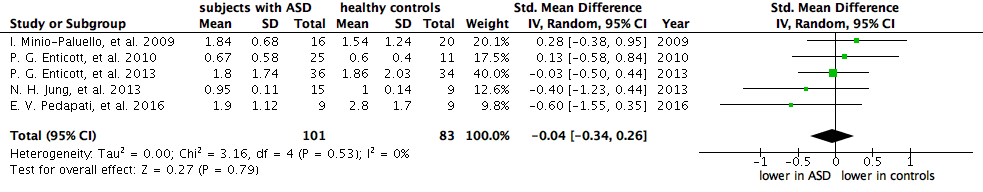

Supplement: Supplementary file 4 — Supplementary Figure 2. [file 41398_2019_444_MOESM4_ESM.docx]

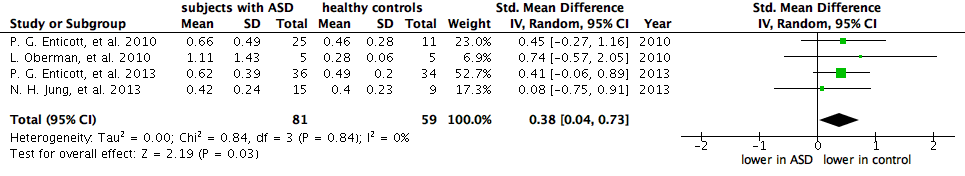
**Supplementary Figure 3.** Group differences in SICI between individuals with ASD and controls.

Supplement: Supplementary file 5 — Supplementary Figure 3. [file 41398_2019_444_MOESM5_ESM.docx]

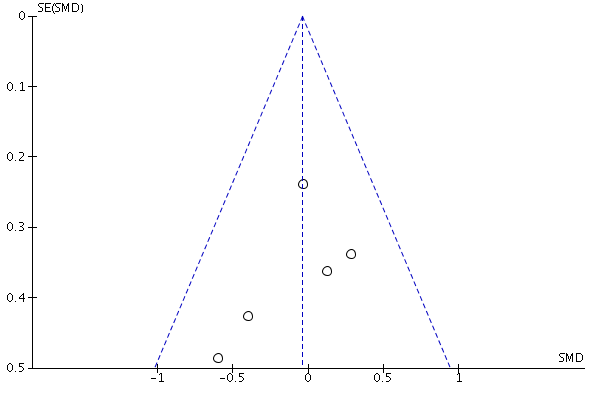
**Supplementary Figure 6.** A funnel plot for MEP data in the included studies

Supplement: Supplementary file 8 — Supplementary Figure 6. [file 41398_2019_444_MOESM8_ESM.docx]

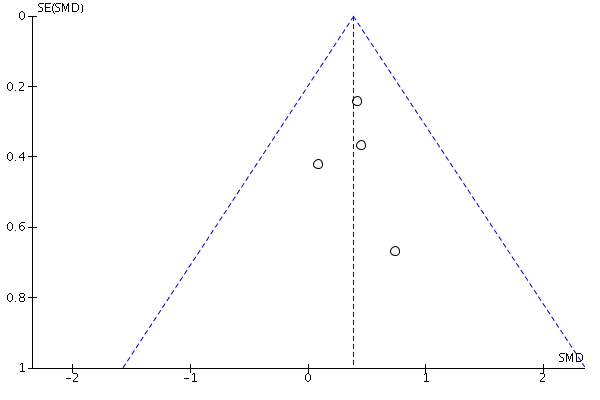
**Supplementary Figure 7.** A funnel plot for SICI data in the included studies

Supplement: Supplementary file 9 — Supplementary Figure 7. [file 41398_2019_444_MOESM9_ESM.docx]
