# Supplementary material for: Motor cortex excitability and inhibitory imbalance in autism spectrum disorder assessed with transcranial magnetic stimulation: a systematic review
Source: Transl Psychiatry. 2019 Mar 7;9:110. doi: 10.1038/s41398-019-0444-3 (PMC6405856; doi:10.1038/s41398-019-0444-3)
Supplement: Supplementary file 7 — Supplementary Figure 5. [file 41398_2019_444_MOESM7_ESM.docx]

**Supplementary Figure 5.** Meta-regression analysis examining the relationship among male ratio and MEP amplitudes between individuals with ASD and controls.
